# Leu72Met polymorphism of *GHRL* gene decreases susceptibility to type 2 diabetes mellitus in a Mexican population

**DOI:** 10.1186/s12902-020-00596-3

**Published:** 2020-07-22

**Authors:** Edgar Alfonso Rivera-León, Mara Anaís Llamas-Covarrubias, Sergio Sánchez-Enríquez, Erika Martínez-López, Mercedes González-Hita, Iris Monserrat Llamas-Covarrubias

**Affiliations:** 1 Departamento de Biología Molecular y Genómica, Universidad de Guadalajara, Centro Universitario de Ciencias de la Salud, CUCS, Postal adress: Sierra Mojada 950, Colonia Independencia, CP, 44340 Guadalajara, Jalisco Mexico; 2 Departamento de Clínicas, Universidad de Guadalajara, Centro Universitario de los Altos, Tepatitlán de Morelos, Jalisco Mexico

**Keywords:** Type 2 diabetes mellitus, Ghrelin, Polymorphism, Leu72Met

## Abstract

**Background:**

Type 2 diabetes mellitus (T2D) is the most frequent type of diabetes. It has a multifactorial etiology, affecting millions of people worldwide. Ghrelin gene (*GHRL*) encodes the ghrelin peptide, which promotes food intake, induces body weight and adipogenesis. Several single nucleotide polymorphisms (SNPs) in *GHRL* gene have been associated with metabolic diseases. A protective effect of the Leu72Met (rs696217) polymorphism has been described for T2D in some populations, but this effect seems to depend on the ethnicity of the patients studied.

**Methods:**

The aim of this study was to investigate the association between the *GHRL* Leu72Met (rs696217) SNP with the development of T2D and serum ghrelin levels in a Western Mexican population. We performed a case-control study in which we included 284 subjects (159 with previous T2D diagnosis and 125 control subjects (CS)). Leu72Met SNP was genotyped by using PCR-RFLPs technique. Serum ghrelin levels were measured using a commercial enzyme immunoassay. Genotypic and allelic distributions were compared using Chi square test. Student T-test and Mann-Whitney U test were used to compare quantitative variables. Odds ratio (OR) was used to evaluate the association between alleles or genotypes and T2D. Multiple and logistic regression models were performed for adjustment. A two-tailed *p*-value ≤0.05 was considered statistically significant.

**Results:**

Leu72Leu genotype was more frequent among T2D compared to CS (*p* < 0.05). After adjusting for age and body composition, there was a significant protective effect of the 72Met allele for T2D development (OR 0.40 IC 95% 0.23–0.70; *p* ≤ 0.001). Fasting serum ghrelin levels were lower in T2D than CS (*p* ≤ 0.0001) irrespective of age, body weight and BMI. No associations were found between genotypes and ghrelin serum levels in our population.

**Conclusions:**

The *GHRL* 72Met allele decreases susceptibility for T2D development in a Western Mexican population. Serum ghrelin levels are lower in T2D independently of Leu72Met polymorphism genotype.

## Background

Diabetes mellitus is an heterogeneous group of disorders characterized by hyperglycemia due to an absolute or relative deficit in insulin production or action [1]. Type 2 diabetes mellitus (T2D) is the most frequent metabolic alteration accounting for around 90% of all diabetes cases [2]. Worldwide, the International Diabetes Federation (IDF) estimated that there were 415 million adults had diabetes mellitus patients in 2015, and predicted a rise to 552 million by 2030 [3, 4]. In Mexico, the prevalence of T2D is 13.9% in adult population [5] and also it is also predicted to increase [6], given the lifestyle risk factors of Mexican population [7–9]. Its etiology is multifactorial involving a sedentary lifestyle, obesity, poor quality of diet and genetic factors [3]. T2D courses with a progressive deterioration of pancreatic beta cells, leading to chronic hyperglycemia that conducts in most cases to organ failure such as kidney, liver, retina, nervous and cardiovascular system [10]. Ghrelin gene (*GHRL*) encodes ghrelin peptide, a 28 amino acids hormone produced mainly in the stomach that is involved in food intake regulation. Ghrelin is an orexigenic hormone that promotes food intake, induces body weight gain and adipogenesis. This hormone is recognized by the growth hormone secretagogue receptor 1a (GHSR1a) which is present in various tissues such as pituitary, myocardium, pancreatic islets and in the hypothalamus. Once the interaction with its receptor is stablished, ghrelin mainly induces the expression of food intake stimulating the neuropeptides: neuropeptide Y and Agouti related peptide and inhibits the expression of proopiomelanocortin, an anorexigenic neuropeptide. Additional functions have also been reported for ghrelin such as: modulation of food reward, olfactory sensitivity, myocardial contraction, sleep, stress and depression regulation [11, 12]. Several polymorphisms exist in the ghrelin gene and some of them seem to be associated with metabolic diseases [12]. In particular, the Leu72Met (rs696217) polymorphism shows a protective effect to T2D in some populations [13, 14]. The Leu72Met polymorphism consists of a transversion of an Cytosine for an Adenine in the position 247 of the *GHRL* gene, consequently leading to an amino acid change (missense) from Leucine to Methionine in codon 72 [15]. It is known that there is a differential effect of this polymorphism in terms of T2D susceptibility among different ethnic groups [16], so far, there is no evidence about genotypic and allelic frequencies and their association with T2D and in serum ghrelin levels in Mexican population.

## Methods

### Subjects

A case-control study in which were included one hundred and fifty-nine adult subjects with previous diagnosis of T2D and one hundred and twenty-five adult normal weight control subjects (CS) that did not present T2D was conducted. We included male and female participants of age ≥ 30 in both groups, and in the CS group we also considered a BMI not higher than 24.9 as inclusion criteria. None of them had history of cancer, rheumatologic, kidney or liver disease, or thyroid and parathyroid disorders or were using medication that interfere with glucose tolerance or serum lipid levels. This study was approved by the local ethics committee (registration code: C.I. 086) and all the participants signed a written informed consent in accordance with the Declaration of Helsinki.

### Anthropometric measurements

Height was measured using a stadiometer (SECA Inc., México). Waist and hip circumferences were obtained in centimeters by a measuring tape (SECA 201). Body mass index (BMI; kg/m2), weight and body fat percentage (BFP) were obtained using a bioelectrical impedance scale (TFB-300A, Tanita®, Tokyo, Japan). All anthropometric measurements were performed in accordance to the International Society for the Advancement of Kinanthropometry standards.

### Biochemical analysis

Blood samples were obtained by venipuncture with Vacutainer® system from all subjects after an 8 h overnight fasting period. Serum levels of fasting glucose (FG), triglycerides (TG), total cholesterol (TC) and high-density lipoprotein cholesterol (HDL-C) were determined using standard biochemical methods (BioSystems®, Barcelona, Spain). In addition, a subsample of the study individuals was made in order to measure total plasma ghrelin levels in duplicate with the RayBiotech, Inc., Norcross GA, USA enzyme immunoassay.

### Genotyping

DNA isolation was performed by the Miller method [17]. Samples of DNA of each subject were analyzed by the polymerase chain reaction of restriction fragment-length polymorphisms (PCR-RFLPs) in order to determine the Leu72Met genotype. First, a PCR was carried out to amplify the region of the SNP using the following primers: forward 5′ TCTCTGGGCTTCAGTCTTCT 3′ and reverse 5′ CACTGCCACCTCTCCTGC 3′. PCR was performed in a final volume of 25 μL, containing 200 ng of gDNA, 20 μM of each primer, 1.5 U/μL of Taq polymerase (Fermentas Thermo Fisher Scientific®, Waltham MA, USA), 1X buffer, and 0.1 mM of each dNTP (Dongsheng Biotech Co., Guandong, China) with the next conditions: denaturation (95 °C 30 s), annealing (60 °C 30 s) and extension (72 °C 30 s) for 30 cycles performed on a programmable thermocycler (TC-300, Techne®, Staffs, UK). Finally, 10 μL of the amplified fragments by PCR were incubated for enzyme digestion with 1.5 U of *BsrI* restriction enzyme (New England Biolabs®, Ipswich MA, USA) for 20 min at 60 °C. PCR fragments and digestion products were analyzed in 2% agarose gel stained with Gel Red™ (Biotinum, Inc., Hayward CA, USA).

### Statistical analysis

The significance of genotypic and allelic frequencies differences was compared using Chi square test. Student T-test and Mann-Whitney U test were used to compare quantitative variables according to their distribution. The association between genotypes or alleles with T2D was analyzed with Odds ratio (OR). Also, there was performed multiple and logistic regression models for the adjustment of ghrelin serum levels and genotype. It was considered as statistically significant when a two-tailed *p*-value ≤0.05 resulted. All statistical analyses were done by means of IBM SPSS 20.0 (SPSS, Inc., Chicago, IL, USA) and GraphPad Prism 8 (GraphPad Software®, La Jolla CA, USA).

## Results

**Fig. 1:**
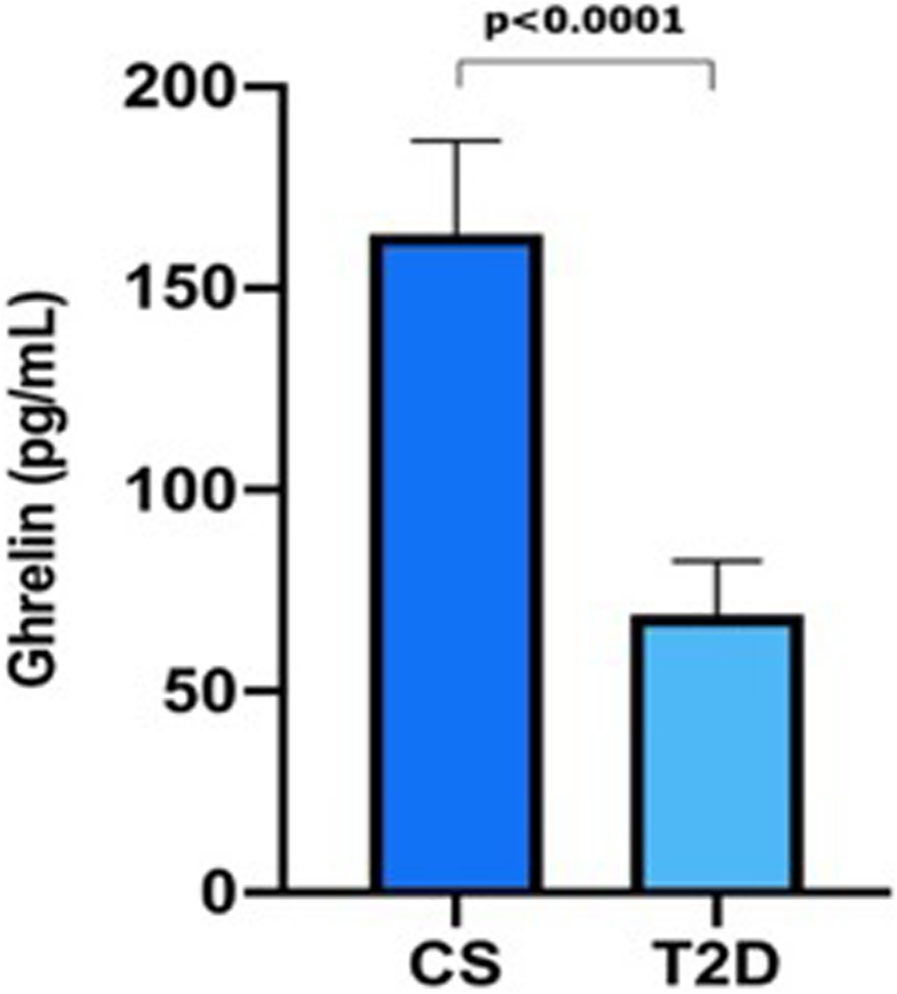
Serum ghrelin levels among study groups. CS control subjects (*n* = 24). T2D Type 2 Diabetes Mellitus group (*n* = 65). Values are expressed as median (interquartile range), adjusted using the multiple regression analys. Figure created with GraphPad Prism 8 software

Basal characteristics of the study groups are shown in Table 1. T2D group showed a higher age and also significantly higher values in all clinical variables and anthropometrical variables than CS as expected. Ghrelin serum levels were evaluated in a subsample of 89 individuals (65 from T2D group and 24 CS) and we found significantly lower levels in serum T2D than in CS being: 50.5(60.8) pg/mL and 177.6(72.8) pg/mL respectively, Fig. 1 (values are expressed as median (interquartile range) *p* ≤ 0.0001). After an adjustment by age, body weight and BMI, the differences in serum ghrelin levels by groups remained significant (p ≤ 0.0001).

**Table 1:** Demographic, clinical and anthropometric characteristics of the study groups

	CS *n* = 125	T2D *n* = 159	p
**Demographic**			
Age (years)	40.3 ± 8.8	51.1 ± 9.0	**≤0.0100**
Gender			
Female n (%)	86 (68.8)	95 (59.7)	NS
Male n (%)	39 (31.2)	64 (40.3)	NS
**Clinical and anthropometric**			
Weight (kg)	63.1 ± 8.7	79.4 ± 15.9	**≤0.0001**
BMI (Kg/m^2^)	23.3 ± 1.6	30.3 ± 5.5	**≤0.0001**
SBP (mmHg)	117.1 ± 13.5	124.2 ± 15.4	**0.0040**
DBP (mmHg)	75.2 ± 8.6	78.4 ± 10.9	**0.0010**
Waist (cm)	80.5 ± 7.7	99.2 ± 11.6	**≤0.0001**
Hip (cm)	98.5 ± 4.9	107.7 ± 11.0	**≤0.0001**
BFP (%)	27.1 ± 6.7	34.1 ± 8.9	**≤0.0001**
Glucose (mg/dl)	89.2 ± 14.5	182.9 ± 77.0	**≤0.0001**
Cholesterol (mg/dl)	194.2 ± 76.9	211.6 ± 54.8	**0.0400**
Triglycerides (mg/dl)	121.9 ± 88.6	209.9 ± 131.2	**≤0.0001**
c-HDL (mg/dl)	50.8 ± 13.7	42.4 ± 14.3	**≤0.0010**
c-LDL (mg/dl)	126.1 ± 45.8	149.4 ± 52.6	**0.0020**

*CS* Control Subjects, *T2D* Type 2 Diabetes Mellitus, *BFP* Body Fat Percentage, *c-HDL* High Density Lipoprotein cholesterol, *c-LDL* Low Density Lipoprotein cholesterol, *NS* non-significant. Values of gender are expressed as frequency (percentage) analyzed by chi square test. All the other variables are expressed as means±SD and compared by Student’s t-test

Genotypic and allelic distributions are shown in Table 2. Both allele and genotype proportions were in equilibrium according to Hardy-Weinberg’s law. Genotypic frequencies were significantly different between study groups (*p* ≤ 0.005). Both the Leu72Leu genotype and Leu72 allele, were markedly more frequent in T2D group than in CS. In the risk analysis, it was detected that the Leu72Met genotype and the 72Met allele were associated with decreased risk for T2D (OR 0.41 IC 95% 0.23–0.70; *p* = 0.0014 and OR 0.52 IC 95% 0.33–0.84; *p* = 0.006 respectively). After adjustment by age, body weight and BMI, Leu72Met genotype was no longer associated with T2D risk (*p* = 0.37), but the 72Met allele remained as a significant protective factor (OR 0.40 IC 95% 0.23–0.70; *p* ≤ 0.001). Serum ghrelin levels did not show differences when compared between genotypes (data not shown).

**Table 2:** Genotypic and allelic distribution in T2D and CS

	**Frequency n (%)**					
**Genotype**	**CS**	**T2D**	**p**	**OR**	**IC95%**	**p**^**b**^
Leu72Leu^a^	79 (63.2)	127 (79.9)		–	–	–
Leu72Met	43 (34.4)	28 (17.6)	< 0.050	0.41	0.23–0.70	NS
Met72Met	3 (2.4)	4 (2.5)		0.83	0.18–3.81	NS
**Allele**						
Leu^a^	201 (80.4)	282 (88.7)		–	–	**–**
Met	49 (19.6)	36 (11.3)	< 0.050	0.40	0.23–0.70	**≤0.001**

*NS* non-significant. Genotypes are in Hardy-Weinberg equilibrium; Data was analyzed by Chi squared test. ^a^Genotype and allele of reference. ^b^Adjusted by age, body weight and BMI using multivariate logistic regression analysis

## Discussion

This study aimed to describe the association between Leu72Met polymorphism and the risk of developing T2D in a Mexican population. We studied two groups, individuals with T2D and individuals with no T2D and with normal weight. As expected, T2D group showed significantly higher values in all clinical and metabolic variables, as compared to CS; this is explained by the natural history of T2D in which one or more of the following signs are common: overweight, obesity, high blood pressure, hyperglycemia and dyslipidemia [3]. On the other hand, lower serum ghrelin levels were detected in the T2D group. Since our groups were different in age distribution and the association between ghrelin levels body composition and age has been previously described [18, 19], we carried out an adjusted analysis in order to exclude the effect of age, body weight and BMI in our results. After adjusting, the difference detected in ghrelin levels in both groups remained significant (*p* ≤ 0.0001). About that, other studies have also encountered lower serum ghrelin levels in diabetic patients [20–22]. Given these results, it is possible that somehow T2D modifies ghrelin production. Tong J et.al., showed that the continuous infusion of ghrelin in healthy volunteers induced the suppression of glucose stimulated-insulin secretion [23]; in line with that, other studies have demonstrated that ghrelin increases plasma glucose levels and decreases fasting insulin levels [12]. A study by Sun Y et.al., were ghrelin gene was deleted in ob/ob mice showed a reduction in hyperglycemia and enhancement in glucose-induced insulin secretion [24]. It is possible that in T2D, lower ghrelin serum levels are produced by a compensatory mechanism that seeks to avoid the increment in circulating levels of glucose and those levels are independent of the effect of Leu72Met genotype.

Regarding the Leu72Met polymorphism, genotypic and allelic distributions of this study are in accordance to what has been reported before by Berthold HK, Bing C, Zavarella S in Caucasians and by Kim S in Asians [13, 25, 26]. When we compared genotypic and allelic frequencies between groups, both Leu72Leu genotype and 72Leu allele were a lot more frequent in T2D than in CS. The association analysis with T2D, showed that 72Met allele and Leu72Met genotype were protective against T2D, but after the adjustment, only the 72Met allele remained associated, suggesting that the protective effect of Leu72Met is given by 72Met allele. To this respect, it has been described that the 72Met allele induces protection against T2D in Caucasians [13], whereas the opposite occurs in Asiatic and Danish populations [14, 25, 27, 28]. Moreover, the 72Met allele has also, been associated as a protective against insulin resistance and body fat accumulation [26, 29]. Finally, the 72Met allele has been associated with BMI increase and early development of obesity in Japanese adults and in Italian adolescents [30, 31]. All of this evidence contributes to the controversial role of this polymorphism in T2D, where its effects in susceptibility varies in different ethnic groups as it has also been described for other human features. It is known that in humans, approximately 15% of the SNP’s are population-specific and the differences could be due to the different proportions of SNP alleles in a specific population [16, 32]. But at the moment, the underlying mechanisms of molecular evolution of this SNP between different populations is unclear. Our results are the first evidence about the relationship of this polymorphism and T2D susceptibility in Mexican population. However, this data should be interpreted with caution given the limited sample size of the study. There is a need of more studies for a better elucidation of the relation of genetic variations of the ghrelin gene with metabolic function in this population.

## Conclusions

72Met allele of the Leu72Met polymorphism of *GHRL* gene decreases the risk of T2D development in Mexican population. Serum ghrelin levels are decreased in T2D patients as compared to CS independently with the Leu72Met polymorphism genotype in a Mexican population.

## Data Availability

The datasets used and/or analyzed during the current study are available from the corresponding author on reasonable request at the following e-mail: irism.llamas@gmail.com.

## Abbreviations

T2D: Type 2 Diabetes Mellitus
IDF: International Diabetes Federation
*GHRL*: Ghrelin Gene
GHSR1a: Growth hormone secretagogue receptor
CS: Control Subjects
BFP: Body Fat Percentage
FG: Fasting Glucose
TG: Triglycerides
TC: Total Cholesterol
DNA: Deoxyribonucleic acid
PCR: Polymerase Chain Reaction
RFLP: Restriction Fragment Length Polymorphism
SNP: Single Nucleotide Polymorphism
HDL-C: High Density Lipoprotein
OR: Odds Ratio
